# Low parasite connectivity among three malaria hotspots in Thailand

**DOI:** 10.1038/s41598-021-02746-6

**Published:** 2021-12-02

**Authors:** Hsiao-Han Chang, Meng-Chun Chang, Mathew Kiang, Ayesha S. Mahmud, Nattwut Ekapirat, Kenth Engø-Monsen, Prayuth Sudathip, Caroline O. Buckee, Richard J. Maude

**Affiliations:** 1 Institute of Bioinformatics and Structural Biology and Department of Life Science, National Tsing Hua University, Hsinchu, Taiwan; 2 Department of Epidemiology and Population Health, Stanford University, Stanford, CA USA; 3 Department of Demography, University of California, Berkeley, USA; 4 Mahidol-Oxford Tropical Medicine Research Unit, Faculty of Tropical Medicine, Mahidol University, Bangkok, Thailand; 5 Telenor Research, Oslo, Norway; 6 Division of Vector Borne Diseases, Ministry of Public Health, Nonthaburi, Thailand; 7 Harvard TH Chan School of Public Health, Harvard University, Boston, USA; 8 Centre for Tropical Medicine and Global Health, Nuffield Department of Medicine, University of Oxford, Oxford, UK

**Keywords:** Malaria, Computational biology and bioinformatics

## Abstract

Identifying sources and sinks of malaria transmission is critical for designing effective intervention strategies particularly as countries approach elimination. The number of malaria cases in Thailand decreased 90% between 2012 and 2020, yet elimination has remained a major public health challenge with persistent transmission foci and ongoing importation. There are three main hotspots of malaria transmission in Thailand: Ubon Ratchathani and Sisaket in the Northeast; Tak in the West; and Yala in the South. However, the degree to which these hotspots are connected via travel and importation has not been well characterized. Here, we develop a metapopulation model parameterized by mobile phone call detail record data to estimate parasite flow among these regions. We show that parasite connectivity among these regions was limited, and that each of these provinces independently drove the malaria transmission in nearby provinces. Overall, our results suggest that due to the low probability of domestic importation between the transmission hotspots, control and elimination strategies can be considered separately for each region.

## Introduction

Malaria incidence in Thailand has decreased significantly in recent years^1,2^. As the number of cases has decreased with ongoing elimination efforts, the number of transmission foci has greatly reduced with persistent foci now principally in and around forest areas along international borders. The incidence is generally higher on the other side of these international borders and they are all relatively porous with high potential for importation across them^3^. Of particular concern is the potential for importation and subsequent spread of artemisinin and ACT (artemisinin-based combination therapy) resistant *P. falciparum*. Failure of these parasites to respond to treatment and onward transmission in areas that have reduced or eliminated malaria may lead to re-establishment of the disease which could derail progress towards the elimination target for all malaria of 2024^4^. There are three principal malaria endemic regions in Thailand, the northeast bordering Lao PDR and Cambodia (particularly Ubon Ratchathani and Sisaket provinces), west bordering Myanmar (particularly Tak province), and south bordering Malaysia (particularly Yala province).

As transmission intensity decreases, mapping spatial heterogeneities in transmission and identifying regional sources and sinks of infections becomes increasingly important for resource allocation to achieve elimination efficiently^5,6^. The national strategic plan for malaria elimination in Thailand^4^ relies on interrupting local transmission and preventing reimportation. Interventions include using the 1-3-7 strategy for surveillance and response^7,8^, distribution of long-lasting insecticide treated bed nets and indoor residual spraying. Responsibility for public health activities is gradually being decentralized to 13 health regions with particular functions managed at community level^9^. National coordination is led by the Division of Vector Borne Diseases. Essential to planning and coordinating elimination activities across the country is an understanding of the degree to which domestic importation may occur between different endemic areas. However, information on connectivity between these areas has been lacking.

Multiple approaches can be used to estimate malaria connectivity, including quantifying genetic similarity of parasites, collecting travel surveys and performing case investigation, and modeling how human movements impact the spread of malaria parasites^6^. Previous studies, using genetic data, have found that genetic lineages of *P. falciparum* parasites differed between samples collected in these three regions, and case investigation suggested imported *P. falciparum* cases from nearby countries^10^. More recently it has been established that the different populations of *P. falciparum* in these areas also have differing degrees of antimalarial resistance. In the east, a single artemisinin resistant PfPailin Cys580 Tyr haplotype predominates with increasing prevalence of piperaquine resistance associated with pfplasmepsin2 gene amplification and mutations in pfcrt^11^. This variant has likely spread from Cambodia as part of a selective sweep, as it has to parts of Lao PDR and Vietnam. In the west of Thailand, and across the border in Myanmar, genetic diversity is greater with multiple haplotypes and less frequent resistance^11^. In the south of Thailand, artemisinin resistance is virtually absent and lineages are again different^11^ from other regions in Thailand. Similarly, genetically distinct populations of *P. vivax* have been identified in Tak and Ubon Ratchathani^12^. While genetic data suggest differentiation among different epidemic regions, genetic data might be influenced by sampling bias and other data types have not been used to provide independent information on the importance of domestic importation on *P. falciparum* transmission in Thailand.

Here, we developed a metapopulation model of malaria transmission to quantify parasite flow across the country. We parameterized this model using mobile phone call detail records (CDR) from 11 million subscribers over three months to infer human movements between provinces and constructed a metapopulation model to quantify parasite flow across the country.

## Materials and methods

### Incidence and population size data

All suspected cases visiting malaria clinics (MCs), malaria posts (MP) and hospitals (mainly government hospitals) were confirmed by either rapid diagnostic test (RDT) or microscopy (thick or thin film). Information on confirmed cases were routinely gathered and entered into a national surveillance system by the data management unit of each health facility. Malaria incidence records were directly reported to the Ministry of Public Health and stored at the Bureau of Vector Borne Diseases (BVBD—now Division of Vector Borne Diseases (DVBD)) database. Individual information of each patient such as demographics, place of residence, place of diagnosis, treatment received, diagnostic method and date of attendance were pooled and the completeness of the data verified by BVBD. Data obtained for this study was from 2012 through 2017 and individual patient records were aggregated at province level by BVBD prior to analysis. Annual Population data were obtained from the Ministry of Interior (MOI).

### Mobile phone data

Anonymised, aggregated call detail record (CDR) data were obtained in partnership with Telenor Research and DTAC, and includes 11 million subscribers using a DTAC SIM card between August 1 and October 20 of 2017. We estimated the daily movement of individuals between all pairs of provinces based on the CDR data using previously developed algorithms^13^.

### Metapopulation model

Following Chang et al.^21^, we constructed a metapopulation model to estimate parasite flow between locations (admin level 1, province) and the proportion of domestically imported or local cases. Daily human movements between locations (*T*_*ij*_) were estimated based on the CDR data^13^. We assumed that subscribers who did not change their locations or did not use their phones stayed in the same location.

The risk of infection in each location was assumed to be proportional to its incidence (*I*_*i*_) as determined using residential addresses of reported cases. The proportion of cases in location *i* from different locations *j* (*P*_*ij*_) was proportional to the product of the number of trips to each destination and its incidence (Eq. (1)).

1 $$P_{ij} = \frac{{T_{ij} I_{j} }}{{\mathop \sum \nolimits_{j}^{{}} T_{ij} I_{j} }}$$

We did not consider international importation in this model, and therefore the total proportion of domestically imported cases was equal to 1–*P*_*ii*_. Parasite flow between locations (*M*_*ij*_, the number of cases in location *i* from location *j*) was calculated by Eq. (2):

2 $$M_{ij} = N_{i} I_{i} P_{ij} ,$$

where *N*_*i*_ is the population size of location *i*. Source score (*S*_*i*_) and sink score (*R*_*i*_) for each location were calculated from *M*_*ij*_ as follows:

3 $$S_{i} = \mathop \sum \limits_{j \ne i}^{{}} M_{ji} \quad {\text{and}}\quad R_{i} = \mathop \sum \limits_{j \ne i}^{{}} M_{ij} .$$

We compared the metapopulation model parameterized by the mobile phone calling data with a standard diffusion model (gravity model^14^) that is often used in the absence of mobility data. Since empirical travel data other than the mobile phone calling data were not available for model fitting, we simply assumed that human movements increased with population sizes of both locations and decreased with the geographic distance between them in the gravity model. Because this relationship can only be used between locations, we used the probability of staying in the same location estimated from the mobile phone calling data in the gravity model.

### Ethical approval

This study protocol was approved by the Ethics Committee for Research in Human Subjects, Department of Disease Control, Ministry of Public Health, under the Royal Thai Government. The protocol number is 1/60-001, FWA 00013622. The analysis used only anonymized, aggregated routinely collected malaria surveillance data reported to the Ministry of Public Health and the ethical committee of the Department of Disease Control, Ministry of Public Health, Thailand agreed that informed consent was not required. All methods were carried out in accordance with the relevant guidelines and regulations.

## Results

**Figure 1:**
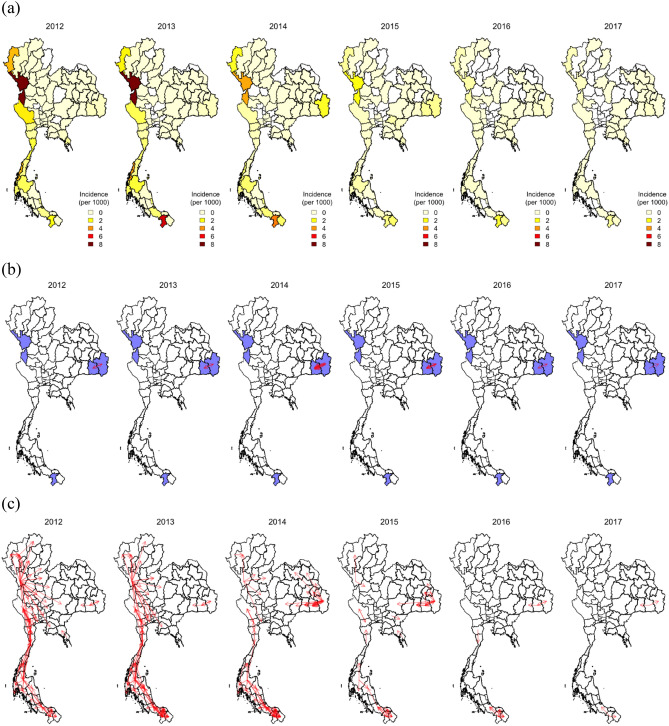
Spatial and temporal case data and estimated parasite flow. (**a**) Spatial distribution of malaria case data from 2012 to 2017. (**b**) Parasite flow among three malaria endemic regions in Thailand (Ubon Ratchathani [Northeast], Sisaket [Northeast], Tak [West], Yala [South] provinces were labeled in blue). (**c**) Parasite flow among all provinces in Thailand. The thickness of the line is proportional to the level of parasite flow. Only parasite flow greater than 1 was plotted.

We examined the spatiotemporal changes in *P. falciparum* malaria incidence from 2012 to 2017 in Thailand (Fig. 1a). While the incidence in Tak Province decreased through time, the incidence in Ubon Ratchathani and Sisaket increased temporarily due to an outbreak along the border in 2014 and 2015. To investigate whether the increase in incidence in Ubon Ratchathani and Sisaket was seeded by domestic importation from either Tak or Yala Province, we inferred parasite flow between these locations based on a metapopulation model parameterized by the mobile phone CDR data. We found that, in contrast to the baseline gravity model (Supplementary Figure S1), malaria infections in these three regions were not directly connected between 2012 and 2017 (Fig. 1b). This is consistent with the mobility comparison between the CDR data and gravity model (Supplementary Figure S2), where the gravity model suggests higher amounts of long-distance travel than observed from the CDR data.

Tak and Yala Provinces were indirectly connected by Provinces between them in 2012 and 2013, and these connections became weaker as the incidence became lower (Fig. 1c). The overall connectivity between all the locations across the country showed that the major source of infection shifted from the West to the Northeast and the South (Fig. 1c and Supplementary Figure S3a), and the Northeast was not directly or indirectly connected with the other two endemic regions during these years.

The amount of domestically imported cases (sink scores) was higher if a location was near any of the three endemic regions and decreased through time (Supplementary Figure S3b). The proportion of domestically imported cases was high in these locations with low incidence (Supplementary Figure S3c), suggesting limited local onward transmission after importation.

## Discussion

We quantified human movements using mobile phone CDR data and used this to inform a metapopulation model to infer temporal patterns of parasite flow between all provinces across Thailand. We found that malaria connectivity between three endemic areas in Thailand was low and decreased as the overall incidence decreased. Our results suggest that the malaria outbreak in Ubon Ratchathani in 2014 and 2015 was unlikely to have been seeded by the two other endemic regions in the previous year. The major source of infection shifted from the West to the Northeast and the South, but the impact of this shift was limited to their nearby locations, and independent from each other, suggesting that malaria control measures in the three main regions can be considered separately. As the country approaches elimination, this supports the WHO-recommended strategy to design different packages of optimal targeted interventions for each area to achieve maximum impact with limited available resources^15^. If the foci were highly connected then such targeting would not be justified.

Our results from the model based on the mobile phone CDR were consistent with parasite genetic studies that showed different parasite lineages between the three endemic areas, and is in contrast to the results based on the gravity model that showed higher parasite flow between the three endemic regions. This suggests that the detailed human movement patterns inferred from the mobile phone CDR were critical in assessing parasite source and sink dynamics in Thailand, and relying on diffusion models could lead to misleading results.

Our approach identified parasite flow and its change over time, but cannot be used to identify which cases were domestically imported and which cases were local. Individual level inference still requires case investigation. However, knowing connectivity in malaria transmission between locations is important for planning for malaria elimination. Our results suggest that elimination plans can consider these three regions independently. They provide reassurance that the current predominant DHA-piperaquine-resistant *P. falciparum* strain in the northeast can be appropriately managed with the current strategy of a different first line treatment than elsewhere in the country. The results further suggest that future importation from a neighboring country is unlikely to lead to substantial onward spread to the other endemic regions.

Human mobility data, together with epidemiological modeling, have been used to understand malaria transmission in several countries. For example, a study in Namibia showed that incorporating human mobility in a model helped reveal heterogeneity of transmission intensity between regions^16^. Wesolowski et al.^17^ utilized CDR data to infer source and sink dynamics for malaria transmission in Kenya. Human mobility data have also been used in other countries, such as Brazil, Bioko Island, and Bangladesh^18–22^ to understand malaria transmission. Our work adds additional support that this kind of approach can provide insights into malaria transmission and be used for guiding control and elimination strategies. There is also potential that the use of CDR and surveillance data in defining sources and sinks of malaria can provide insights on finer spatial scales, and this remains to be explored.

This analysis has some limitations. The CDR data used were from a single company (DTAC) from three months in a single year (2017) and were from the entire population of around 25 million users. Thus, there may be some difference in travel patterns between 2017 and previous years (2012–2016) and between seasons, and the data may not fully reflect the particular travel patterns of people with malaria. However, there were no major political or socio-economic events in that year that should cause unusual travel patterns. Also, the CDR data used in this study is not able to capture travel across international borders. Potentially, this can be improved by combining the CDR data with parasite genetic data from both sides of the border, or methods like travel surveys, or the results of case investigation. These limitations are unlikely to affect the conclusion of low connectivity among three hotspots as areas in neighbouring countries adjacent to the 3 Thai transmission hotspots are remote, in separate countries, and the volume of travel between them is very low.

## Supplementary Information

Supplementary Information.

## Data Availability

Access to mobility data is regulated through non-disclosure agreements (NDAs) and data sharing agreements, and cannot be released publicly. All requests for mobile phone datasets should be directed to Kenth Engø-Monsen at Telenor Research. The malaria database data that support the findings of this study are available from the Thai Ministry of Public Health but restrictions apply to the availability of these data, which were used under license for the current study, and so are not publicly available. Data are however available from the authors upon reasonable request and with permission of the Thai Ministry of Public Health. All the estimates are available at https://github.com/hhc-lab/malaria_Thailand.
