# Supplementary material for: Low parasite connectivity among three malaria hotspots in Thailand
Source: Sci Rep. 2021 Dec 2;11:23348. doi: 10.1038/s41598-021-02746-6 (PMC8640040; doi:10.1038/s41598-021-02746-6)
Supplement: Supplementary file 1 — Supplementary Information. [file 41598_2021_2746_MOESM1_ESM.pdf]

Supplementary Figures

(a)

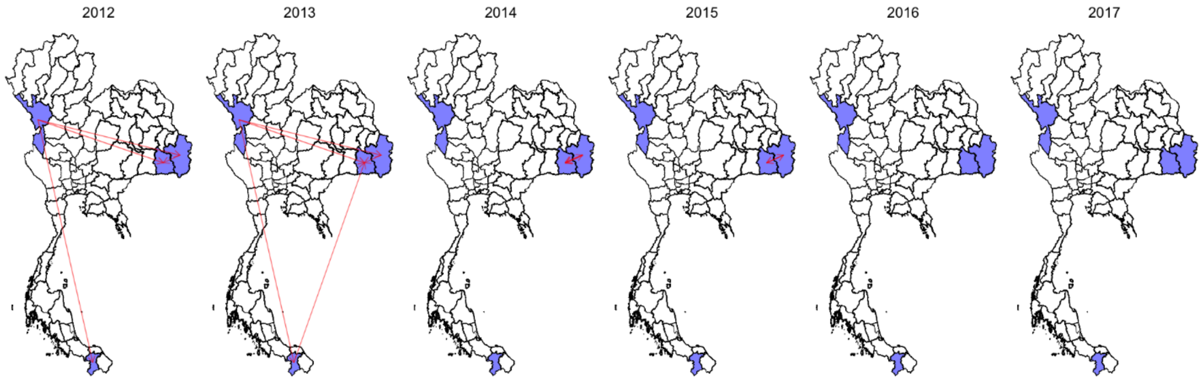

(b)

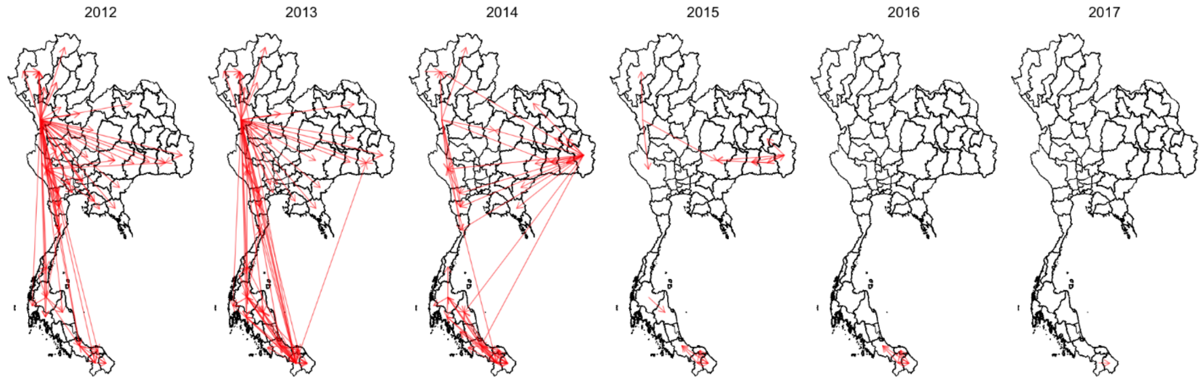

Figure S1. Estimated parasite flow from the gravity model. (a) Parasite flow among three malaria endemic regions in Thailand estimated from the gravity model (Ubon Ratchathani [Northeast], Sisaket [Northeast], Tak [West], Yala [South] provinces were labeled in blue). **(b)** Parasite flow among all provinces in Thailand estimated from the gravity model. The thickness of the line is proportional to the level of parasite flow. Only parasite flow greater than 1 was plotted.

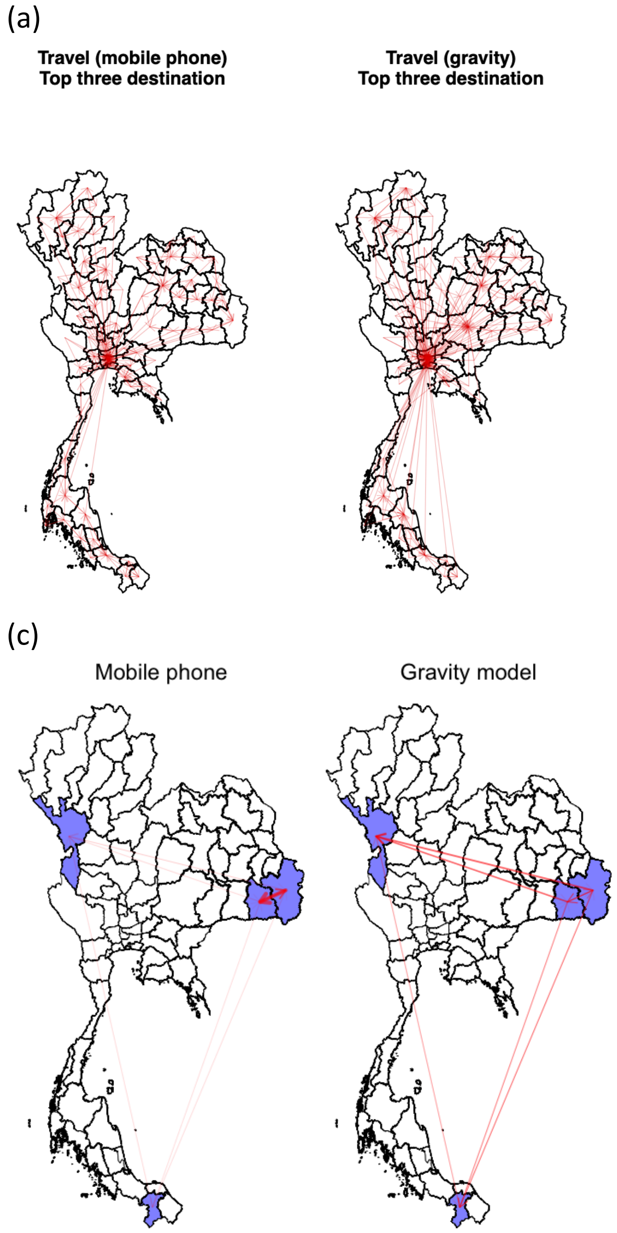

Figure S2. Mobility comparison between the gravity model and the CDR data. Top three destinations **(a)** and origins **(b)** for each province are shown. **(c)** Travel among three malaria endemic regions was shown. The thickness of the line is proportional to the amount of travel in (c).

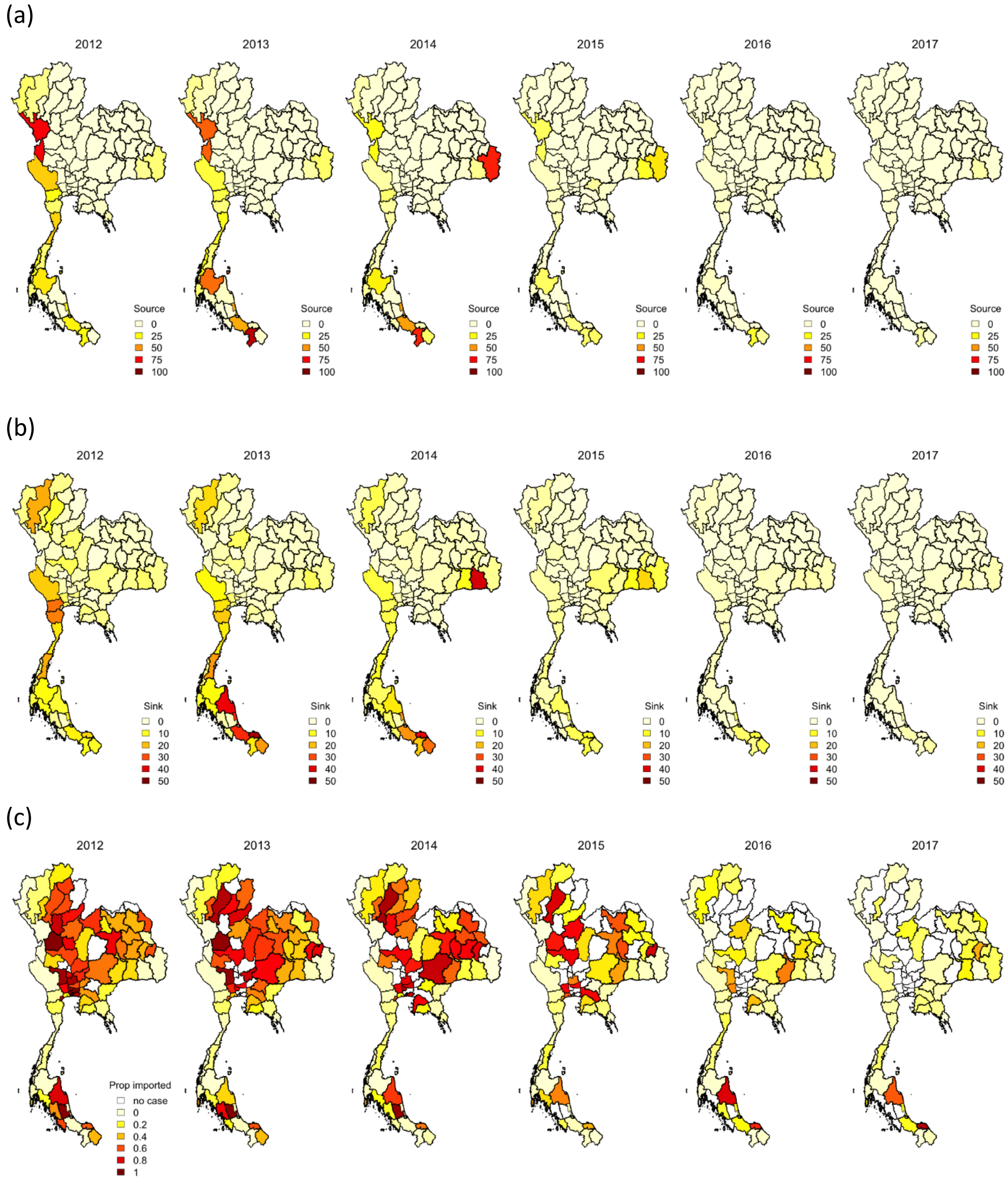

Figure S3. Estimated source score, sink score and the proportion of domestic importation. **(a)** Source score. **(b)** Sink score. **(c)** The proportion of domestic importation.
